# Using Virtual Reality to Assist Students at Academic Risk in Human Anatomy

**DOI:** 10.1007/s40670-025-02291-1

**Published:** 2025-01-29

**Authors:** Michael Foley, Lauren T. Lilley, Lindsay Meyers, Robert Armstrong, Lisa Fore-Arcand, Kelly McCoy, Chad Eitel, Tod R. Clapp, Natascha Heise

**Affiliations:** 1 Macon and Joan Brock Virginia Health Sciences Eastern Virginia Medical School at Old Dominion University, Norfolk, VA USA; 2 Department of Biomedical and Translational Sciences, Macon and Joan Brock Virginia Health Sciences Eastern Virginia Medical School at Old Dominion University, Norfolk, VA USA; 3 Sentara Center for Healthcare Simulation and Immersive Learning, Macon and Joan Brock Virginia Health Sciences at Old Dominion University, Norfolk, VA USA; 4 Department of Psychiatry and Behavioral Sciences, Macon and Joan Brock Virginia Health Sciences Eastern Virginia Medical School at Old Dominion University, Norfolk, VA USA; 5 Continuing Medical Education, Macon and Joan Brock Virginia Health Sciences Eastern Virginia Medical School at Old Dominion University, Norfolk, VA USA; 6 Department of Biomedical Sciences, Colorado State University, Fort Collins, CO USA

**Keywords:** Virtual reality, Medical education, Human anatomy, Academic risk

## Abstract

Learning human anatomy presents a significant challenge for health profession students due to the difficulty in visualizing structures in three dimensions. Virtual reality (VR) has been reported to aid in understanding these relationships. In this study, students at academic risk attended VR sessions alongside their gross anatomy course. Data from post-surveys, observations, and examinations indicated VR participants performed similarly to peers, except in head/neck and pelvis/lower limb exams where performance was lower. Students valued VR for enhancing confidence and understanding. VR shows promise in supplementing anatomy education, particularly for less complex regions, and bridging gaps in traditional learning methods.

## Background

Human anatomy is foundational in medical education for various health profession careers, but students can struggle with the vast amount of content and visualizing spatial relationships [1]. Early academic difficulties can stem from various factors, including educational environment and personal reasons [2, 3], visuospatial ability [4], and access to resources [5]. Peer tutoring has been shown to offer some support, but its impact is inconsistent, especially for students at academic risk [6]. The feeling of information overload may hinder clinical readiness [1], despite the fact that emphasis on clinically relevant anatomy is crucial for critical reasoning in future medical practice [7, 8]. Over the years, anatomy education has employed various resources besides the utilization of cadavers to support and deepen students’ understanding. Time and cost constraints have led to alternative methods such as technology-enhanced learning [9]. Tablet-based applications preceded virtual reality (VR), which offers interactive three-dimensional (3D) models helpful for learning anatomy [10]. VR has shown to aid learners, especially those with lower visuo-spatial ability, by allowing direct manipulation of structures [11, 12]. Using VR, students can study at their own pace addressing various learning needs.

The utilization of VR to visualize anatomical structures has become more common in clinical education [13] with few VR studies focusing on educational implementation [14]. VR is currently prevalent in surgical training and decision-making in neurology [15, 16], cardiothoracic [17, 18], abdominal [19, 20], plastic [21], and orthopedic [22–24] specialties. While VR 3D skills have been proven to be beneficial in residency, preclinical classroom learning still mostly emphasizes 2D information recall [25, 26] creating a disconnect between clinical and didactic learning. In response to the COVID-19 pandemic, online anatomy courses at various institutions were created highlighting the potential of VR in preclinical education [27–29]. VR has been seen as effective as traditional teaching in anatomy when comparing test scores and knowledge gain [30–32]. In a review, spatial ability has been found to affect practical examination performance in identifying cadaver structures [33], suggesting that early VR implementation may be helpful in medical education. Although learners in medicine have reported enhanced satisfaction, motivation, engagement, and perceived usefulness when studying with VR [30, 34, 35], most reported studies involve short, isolated VR sessions [13, 36, 37] and body region complexity and study designs vary, which complicates comparisons [31].

In summary, the literature supports the use of VR in enhancing anatomical understanding, spatial relationships, and transfer of knowledge in clinical years [14, 37]. However, its impact on at-risk students and effective integration in already existing preclinical curricula remain unclear. This study explores the effectiveness of VR as a supplemental tool for health profession students, guided by the following research questions:

1. How does the use of VR in human anatomy affect academically at-risk students’ examination scores?
2. How does the use of VR in human anatomy affect academically at-risk students’ confidence in understanding anatomical spatial relationships?
3. What is the students’ perception of VR as a supplement to their learning in human anatomy?

## Activity

### Human Anatomy Course Structure and Grading

Students (*n* = 123) from Pathologists’ Assistant (PathA, *n* = 20), Physician Assistant (PA, *n* = 88), and Surgical Assisting (SA, *n* = 15) programs at Eastern Virginia Medical School (EVMS) were enrolled in a 16-week human anatomy course of four content blocks of upper limb, head/neck, thorax/abdomen, and pelvis/lower limb. Students visited the cadaver lab twice a week for dissection or study of prosected cadavers with faculty but had 24/7 access to the laboratory. Each block had a written examination of 55 higher order multiple-choice questions, practical examination of 45 identification questions on cadavers, and graded formative quizzes.

### Participants

Students at academic risk identified by remediation status, scoring two standard deviations below the examination average or program director recommendation, were invited to participate in optional VR study sessions. Sixteen students (13.01% of the class) from PathA (*n* = 6, 4.88% of the class and 30% of program cohort), PA (*n* = 9, 7.32% of the class and 10.23% of program cohort), and SA (*n* = 1, 0.81% of class size and 6.67% of program cohort) participated voluntarily beginning at different time points throughout the course. Participants who attended more than one VR session by the end of the semester were asked to participate in the study. Nine completed the post-course survey. Participants included both females (*n* = 7, 77.8%) and males (*n* = 2, 22.2%) ranging in age from 20 to 40 years. Additionally, some students had not taken an anatomy course previously (*n* = 2, 22.2%).

### VR Program Structure

The VR software, developed by Perspectus Technology (Perspectus Inc, Fort Collins, CO, USA), allowed exploration of 3D anatomical models and volumetric imaging (CT/MRI). The software is available for higher education, healthcare, and commercial use. EVMS’ VR setup included VR-capable computers and Hewlett-Packard Reverb head-mounted displays with controllers. Students received an introductory VR lesson at the beginning of each block by faculty and signed up for VR study hours. Thirteen self-directed VR sessions of 2–3 h each were available, including two upper limb, four head/neck, three thorax/abdomen, and four pelvis/lower limb sessions. Faculty assistance during these sessions was limited to technical support to promote self-efficacy and independent learning.

### Data Analysis

This study conducted a mixed method approach of quantitative and qualitative analyses. Data on VR attendance and examination scores were collected. Written and practical examination scores of VR and non-VR groups were compared using unpaired *t*-tests with Welch’s correction. Qualitative data included open-ended survey questions and observational field notes capturing remarks made by participants. An inductive thematic analysis was conducted by three researchers with themes reviewed collectively to ensure reliability and validity. Ordinal data was analyzed using Likert scales (1–5) and statistical analyses were performed using GraphPad Prism 9 (GraphPad Software, Boston, MA, USA) with significance set at *p* = 0.05.

## Results and Discussion

### How Does the Use of VR in Human Anatomy Affect Academically At-Risk Students’ Examination Scores?

**Fig. 1:**
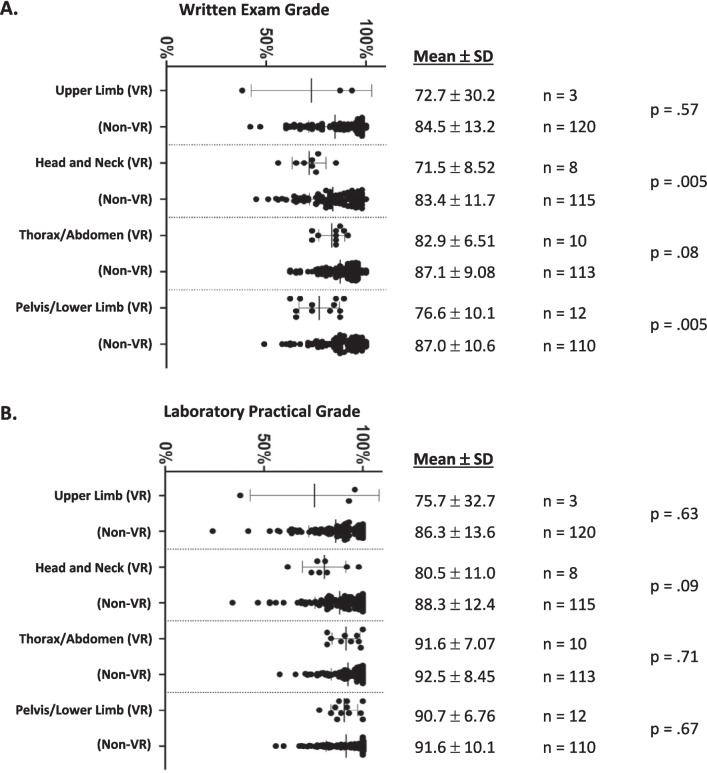
Performance based on using virtual reality (VR) when preparing for the examination (exam). **A** Written examination outcomes. **B** Laboratory practical outcomes. The different examinations covered the upper limb, head and neck, thorax and abdomen, and pelvis and lower limb

Students at academic risk who studied with VR for their written and practical examinations had similar outcomes when compared to the rest of the class with a few exceptions. Mean scores for the groups are shown in a density scatter plot showing the score distributions (Fig. 1). Statistical analysis revealed no significant difference between the mean scores of the VR group compared to the non-VR group for the written examination on upper limb (*p* = 0.57) and thorax/abdomen (*p* = 0.08) and for the practical examination on upper limb (*p* = 0.63), head/neck (*p* = 0.09), thorax/abdomen (*p* = 0.71), and pelvis/lower limb (*p* = 0.67). There was a significant difference between the groups for the written examination on head/neck (*p* = 0.005) and pelvis/lower limb (*p* = 0.005). Similar mean scores for over half of the examinations suggest VR aided student learning in most of the content areas. However, the small sample size of at-risk students (*n* = 3) in the cohort during the upper limb exam limits our ability to draw meaningful conclusions about performance on that assessment. Head/neck and pelvis/lower limb regions have been previously identified as challenging for students [34, 35] indicating VR may be more effective for less complex areas like the thorax, abdomen, and limbs. Similar practical examination performance aligns with the immersive nature of both VR and cadaver laboratories. These results suggest VR implementation can enhance practical examination performance and possibly increase spatial understanding.

### How Does the Use of VR in Human Anatomy Affect Academically At-Risk Students’ Confidence in Understanding Anatomical Spatial Relationships?

**Fig. 2:**
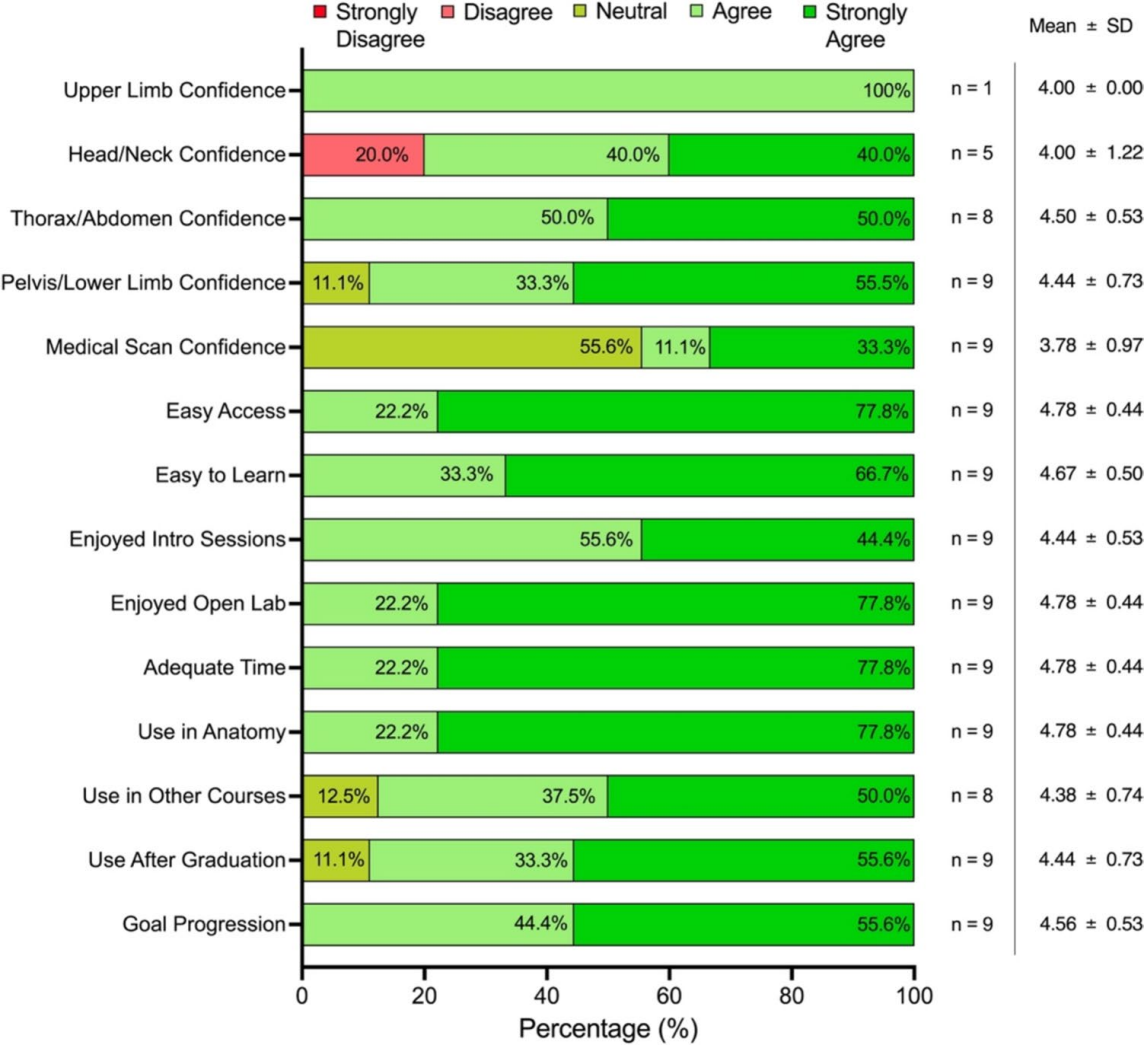
Likert-scale questions from the post-survey. Statistical analysis of the self-perceived feelings of the use of virtual reality (VR). Mean and standard deviation are included on the right of the plot in addition to number of student responses per question (*n*)

Responses to Likert-scale questions showed that most students favored the “agree” and “strongly agree” categories (Fig. 2). Only one student (11.1%) selected “disagree” for VR building confidence in the head and neck. The “neutral” choice was selected by five students (55.5%) for confidence in understanding medical scans, and by one student (11.1%) for confidence in the pelvis/lower limb and for future VR use in other courses and after graduation. The disagreement on confidence levels regarding the head/neck region and neutrality about the pelvis/lower limb was likely due to the perceived complexity and lower examination performance in these areas [34, 35]. Most students felt neutral about their confidence in medical scans, which can be attributed to infrequent use during their sessions and under testing circumstances. However, incorporating medical imaging could potentially improve learning efficiency in understanding spatial relationships [38]. While students appreciated program capabilities, some suggested enhancing detail within the VR program. This contradicts existing research, which suggests that image quality may not significantly impact long-term test performance [39]. Instead, focusing on strategies like retrieval practice through self-quizzing on VR structure labeling could enhance information retention [40].

### What Is the Students’ Perception of VR as a Supplement to Their Learning in Human Anatomy?

Thematic analysis of survey responses and field observations revealed key themes among students (Table 1). They highlighted enhanced “Visualization" of anatomical structures through VR, aiding comprehension beyond cadaveric limitations. “Accessibility” provided additional study flexibility outside scheduled laboratory sessions, which catered to individual learning needs, and the distraction-free “Learning Environment” supported varied study preferences and facilitated focused learning. While students appreciated facility access, they noted functional issues in terms of “Program Functionality” such as missing intricate details and initial VR navigation challenges. Several other studies have addressed the limitations of VR, including challenges with the realism and accuracy of models [41], the increased cognitive load associated with stereoscopic methods [42], and the occurrence of cybersickness during use [43].

**Table 1:** Themes with corresponding subthemes from the open-ended questions with direct student quotes

Question	Main themes	Subthemes	Quotes
Assisted Learning	Visualization	Spatial relationships Making connections Different angles	“immerse [themselves] in the human body” “manipulate in different ways, views, and sizes” “view structures not easily viewed in donors”
	Accessibility		“extra time to learn structures outside of set lab” “take knowledge gained back to lab for further understanding”
Most Liked	Program capabilities	Structure manipulation	“whatever you wanted to make with it”
	Learning environment	Distraction-free Designated study time Study plans Easy access	“wasn’t overwhelming” “study on your own time”
Improvements	Program functionality	Lack of detail Accessibility	“intricate structures needed to know for practical were missing”
	Logistics		“intro sessions when each block starts”
Interaction Frequency	1–2 times per week		
Anatomy Impact	Additional resource	Enhanced learning Spatial relationships	“everyone does not learn the same way” “instead of trying to imagine it on your own”
Tutoring Impact	Positive impact	Various responses	“This is really cool” “I will definitely come back”
Anything Else	Gratitude		“I recommend VR to all students, no matter how they are doing”

Recommendations included improved “Logistics” such as revised introductory sessions and exploring home VR options. Most students preferred weekly VR sessions of 1 to 2 h. Overall, VR was endorsed as a valuable “Additional Resource” for anatomy education, enhancing learning diversity beyond traditional classroom settings. Notably, the students’ unanimous desire to continue using VR for anatomy learning underscores the “Positive Impact” and potential of VR as a valuable tool for enhancing learning engagement.

These feelings reflect other studies in which students felt more engaged in learning anatomy while studying with VR [30, 31, 34, 35]. The positive feedback is further corroborated by the increase in study session attendance throughout the course. The attendance results show promise that students are eager to make time to use VR as a study tool even in the presence of other stressors demanding time throughout professional school [44]. In terms of limitations, VR participants were able to study with other methods and were identified as at-risk of failing so their baseline performance may have been already lower than the average student. A pre-survey could establish baseline perceptions to further caption student perceptions.

To conclude, while traditional dissection remains central in anatomical education [45, 46], the scarcity of cadavers and advancements in VR and other software have reshaped anatomy education [47]. This shift allows students to fully immerse themselves, visualize structures in three dimensions, and deepen their understanding. This exploratory study supports VR as a beneficial supplement to traditional methods, particularly aiding at-risk students for better academic outcomes [48]. The implementation of VR on a smaller scale enhances an established anatomy curriculum, aiming not to replace but to complement cadaver dissection, bolstering student understanding, confidence, and readiness for medical practice.

## Data Availability

The datasets generated and/or analyzed during the study are available from the corresponding author upon reasonable request and approval by the ethics committee.

## Declarations

This manuscript is an original work, has not been published previously, and is not under consideration for publication by any other journal. This also serves as a written permission from all authors to reproduce figures, tables, and questionnaires in both print and electronic forms. All authors have read and accepted the responsibility of the manuscript’s contents.
